# Supplementary material for: Causal Relationship between Aging and Anorexia Nervosa: A White-Matter-Microstructure-Mediated Mendelian Randomization Analysis
Source: Biomedicines. 2024 Aug 16;12(8):1874. doi: 10.3390/biomedicines12081874 (PMC11351342; doi:10.3390/biomedicines12081874)
Supplement: Supplementary file 1 [file biomedicines-12-01874-s001.zip › Supplementary Figure/Figure S4/Singe SNP plot of MR analysis for TL to ubm-b-1768.pdf]

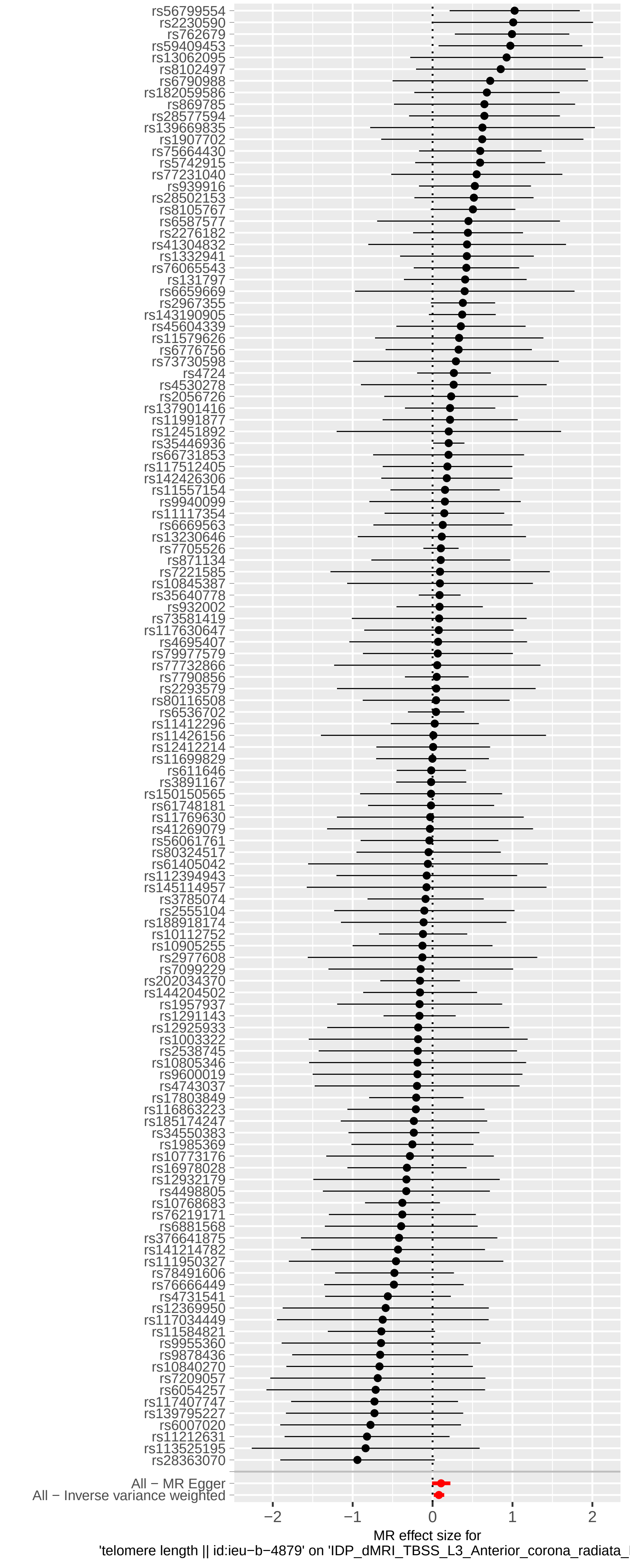
rs56799554
rs2230590
rs762679
rs59409453
rs13062095
rs8102497
rs6790988
rs182059586
rs869785
rs28577594
rs139669835
rs1907702
rs75664430
rs5742915
rs77231040
rs939916
rs28502153
rs8105767
rs6587577
rs2276182
rs41304832
rs1332941
rs76065543
rs131797
rs6659669
rs2967355
rs143190905
rs45604339
rs11579626
rs6776756
rs73730598
rs4724
rs4530278
rs2056726
rs137901416
rs11991877
rs12451892
rs35446936
rs66731853
rs117512405
rs142426306
rs11557154
rs9940099
rs11117354
rs6669563
rs13230646
rs7705526
rs871134
rs7221585
rs10845387
rs35640778
rs932002
rs73581419
rs117630647
rs4695407
rs79977579
rs77732866
rs7790856
rs2293579
rs80116508
rs6536702
rs11412296
rs11426156
rs12412214
rs11699829
rs611646
rs3891167
rs150150565
rs61748181
rs11769630
rs41269079
rs56061761
rs80324517
rs61405042
rs112394943
rs145114957
rs3785074
rs2555104
rs188918174
rs10112752
rs10905255
rs2977608
rs7099229
rs202034370
rs144204502
rs1957937
rs1291143
rs12925933
rs1003322
rs2538745
rs10805346
rs9600019
rs4743037
rs17803849
rs116863223
rs185174247
rs34550383
rs1985369
rs10773176
rs16978028
rs12932179
rs4498805
rs10768683
rs76219171
rs6881568
rs376641875
rs141214782
rs111950327
rs78491606
rs76666449
rs4731541
rs12369950
rs117034449
rs11584821
rs9955360
rs9878436
rs10840270
rs7209057
rs6054257
rs117407747
rs139795227
rs6007020
rs11212631
rs113525195
rs28363070
All − MR Egger
All − Inverse variance weighted
−2
−1
0
1
2
MR effect size for
'telomere length || id:ieu−b−4879' on 'IDP_dMRI_TBSS_L3_Anterior_corona_radiata_
